# Personal and psychophysiological characteristics of the witness experience of cyberaggression in virtual reality

**DOI:** 10.1192/j.eurpsy.2022.580

**Published:** 2022-09-01

**Authors:** G. Soldatova, S. Chigarkova, E. Nikonova, D. Vinitskiy

**Affiliations:** Lomonosov Moscow State University, Faculty Of Psychology, Moscow, Russian Federation

**Keywords:** neuroticism, heart rate variability, virtual reality, cyberaggression

## Abstract

**Introduction:**

The integration of virtual reality into everyday life is changing sociocultural practices, including those related to cyberaggression, which causes negative consequences for mental health and well-being. Particular attention needs to be paid to the poorly researched but widespread roles of bystanders and defenders in cyberaggression (Machackova, 2020; Polanco-Levican, Salvo-Garrido, 2021).

**Objectives:**

The aim is to study the behavioral witness strategies in cyberaggression in VR and their relation to personal and psychophysiological characteristics.

**Methods:**

50 adolescents aged 14-18 years old (50% female) witnessed cyberaggression in an experimental situation in the virtual space of VR-chat. Participants also filled Ten-Item Personality Inventory (Gosling et al., 2003; Egorova, Parshikova, 2016), I7-Impulsiveness (Eysenck, Eysenck, 1985; Kornilova, Dolnikova, 2011), Prosocial Behaviour (Furmanov, Kuhtova, 1998). To determine the functional state Heart rate variability (UPTF 1/30 Psychophysiologist, Mediсom) was measured before and after the experiment.

**Results:**

Behavioral strategies in VR-aggression were divided into uninvolved bystanders (58%) and defenders (42%). All participants experienced stress and functional state decline when faced with cyberaggression, but the defenders were more affected (U=207, p<0.043). Defenders were more likely to have higher social responsibility (U=207, p<0.056) and lower neuroticism (U=208, p<0.054). There were no significant differences in impulsiveness.

**Conclusions:**

Cyberaggression in a virtual environment is stressful, especially for active defenders, who are more included in the situation compared to passive bystanders. The prosocial role of a defender rather than a passive bystander may be related to such characteristics as social responsibility and emotional stability, but not to impulsiveness. The research was supported by RSF (project No. 18-18-00365)

**Disclosure:**

This work was supported by the Russian Science Foundation, project # 18-18-00365.

